# An adult with a finger mass is it benign or malignant?

**DOI:** 10.51866/tyk.148

**Published:** 2022-10-27

**Authors:** Jazlan Jamaluddin, Yeow Siong Lee

**Affiliations:** 1 MD (Moscow), MMed (Family Medicine) (UiTM), Klinik Kesihatan Sauk, Jalan Besar, Lenggong, Sauk, Kuala Kangsar, Perak, Malaysia. Email: jazlanjamaluddin@gmail.com; 2 MBBS (IMU), MMed (Family Medicine) (UM), Klinik Kesihatan Selayang Baru, Jalan Sungai Tua, Batu Caves, Selangor Darul Ehsan, Malaysia.

**Keywords:** Finger, Soft tissue, Neoplasm

## Abstract

We described the case of a 42-year-old man who presented with left index finger mass persisting for 6 months. The mass was small and, painless and had gradually increased in size with limited finger flexion. Physical examination showed a firm mass over the volar surface of the left index finger. There was no tenderness, redness, warmth or punctum. The overlying skin was normal, and the mass did not transilluminate. Further examination of the head and neck, chest, upper limbs and neurovascular system revealed normal findings. No similar masses were found elsewhere in the body. Bedside ultrasound with further investigation and management confirmed the suspected diagnosis.

## Case summary

A 42-year-old man presented with left index finger mass persisting for 6 months. The mass was small and painless and had gradually increased in size with limited finger flexion. No skin changes or neurological symptoms were noted. The mass was not preceded by trauma or fever. There was no other joint swelling, lymph node enlargement, cough, dysphagia or neck swelling. He had a medical history of hypertension managed with telmisartan 40 mg and felodipine 5 mg daily.

**Figure 1:**
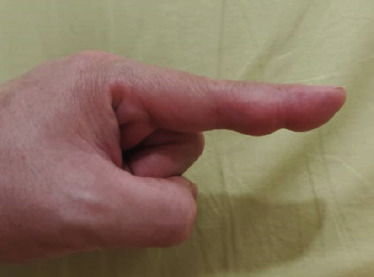
Left index finger showing a mass with no skin changes

**Figure 2:**
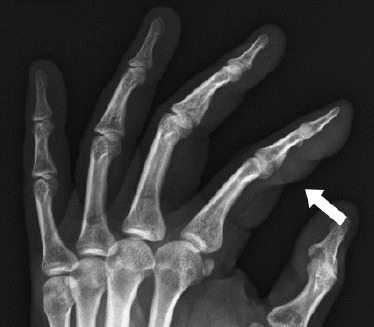
Plain left hand radiograph showing a soft tissue shadow over the index finger with no bony involvement

Physical examination showed a firm, non-fluctuance, non-mobile mass measuring around 0.5×1.0 cm over the volar surface of the left index finger (**Figure 1**). There was no tenderness, redness, warmth or punctum. The overlying skin was normal, and the mass did not transilluminate. The range of movement of both interphalangeal joints especially upon flexion was limited owing to the mass. No effusions were felt. Further examination of the head and neck, chest, upper limbs and neurovascular system revealed normal findings. No similar masses were found elsewhere in the body. Haematological findings, including the full blood count, renal profile, liver function, serum uric acid level, fasting sugar level and lipid level, were within the normal ranges. Plain hand radiography showed a soft tissue shadow with no bony involvement (**Figure 2**).

**Questions**

1. What is the most likely diagnosis?
2. What other initial radiological investigation can be performed?
3. What is the management plan?
4. What are the complications that may arise from this condition?

## Answers

1. The most likely diagnosis is a giant cell tumour (GCT). This is supported by the non-fluctuance, non-mobile nature of the mass. Further, the mass did not transilluminate, which would make the diagnosis of a cyst unlikely. The differential diagnosis for a finger mass includes ganglion cysts, lipomas, accessory muscles, venous or arteriovenous malformations and foreign body granulomas.^1^
2. Bedside ultrasound can be conducted for characterisation of the lesion and demonstration of the relationship with the adjacent tendon.^1^ On ultrasound, GCT usually presents as a nodular tumour-like structure arising against a flexor or extensor tendon. A cyst is typically anechogenic with posterior echo enhancement and no Doppler flow within. Chest radiography can also be performed to identify any lung metastasis.^2^
3. The patient should be referred for crosssectional imaging, such as magnetic resonance imaging (MRI), and further workup to confirm the diagnosis of GCT. Once diagnosis is confirmed, surgical excision is the standard of care for the treatment of GCT.^3^
4. The potential complications include tumour recurrence, neuropathy or limited range of motion and skin necrosis of the finger.^3^

## Case progress

**Figure 3:**
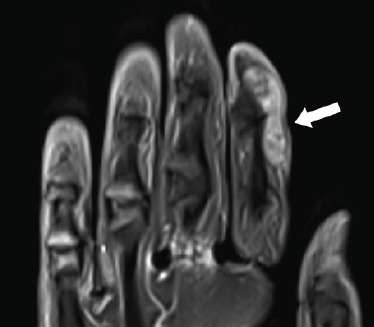
Magnetic resonance image of the left hand showing a solid soft tissue lesion with moderate enhancement post-contrast

The patient was referred to a hand surgeon. Bedside ultrasound of the left index finger mass showed a mixed fluid and solid lesion. Subsequent MRI of the left hand revealed a solid soft tissue lesion sized 0.6×1.2×2.6 cm (**Figure 3**). The diagnosis of GCT of the flexor tendon sheath was highly suspected. Excision of the mass was performed under local anaesthesia. The histopathology examination showed a well-circumscribed lesion composed of mononuclear cells with scattered osteoclast like multinucleated giant cells. Mitotic activity was rare. This confirmed the diagnosis of GCT of the tendon sheath and its benign nature.

## Discussion

GCT of the tendon sheath is the most common form of GCTs. It is also the second most common soft tissue tumour of the hand after ganglion cysts.^3^ GCT accounts for 5% of all primary bone tumours and mostly occurs in women and at the age of 20–40 years.^3^ It mainly affects the meta-epiphyseal area of long bones, although it can also affect the tibia, radius and humerus.^2^ GCTs are usually benign but can be aggressive locally. Bone disruption, especially around the joints, can affect the joint function and mobility.

Diagnostic workup includes a detailed history-taking and physical examination. Plain radiography is beneficial, since GCT can cause erosions in the cortical bone and even invade the medullary space.^3^ Ultrasound can be used to differentiate it from common causes, such as ganglion cysts.^1^ It also offers better spatial resolution than does MRI and is able to identify adhesions and analyse interactions with surrounding tissues using dynamic information about tendon motions and probe pressures. However, MRI is recognised as the most useful diagnostic tool, as it would help with further classification and surgical planning. The treatment of choice for GCT of the tendon sheath is simple excision. However, local benign recurrence of GCT has been reported to occur in 15%–45% of patients.^3^
